# A novel structural modeling magnitude and orientation radiomic descriptor for evaluating response to neoadjuvant therapy in rectal cancers via MRI

**DOI:** 10.1038/s41698-025-01007-3

**Published:** 2025-07-01

**Authors:** Charlems Alvarez-Jimenez, Jacob T. Antunes, Thomas DeSilvio, Zhouping Wei, Marwa Ismail, Joseph E. Willis, Emily Steinhagen, Andrei Purysko, David Liska, Smitha Krishnamurthi, Marka Crittenden, Michael Gough, Kristina Young, Anant Madabhushi, Eduardo Romero, Pallavi Tiwari, Satish E. Viswanath

**Affiliations:** 1 Department of Biomedical Engineering, Case Western Reserve University, Cleveland, OH USA; 2 Computer Imaging and Medical Application Laboratory, Universidad Nacional de Colombia, Bogotá, Colombia; 3 Department of Machine Learning, Moffitt Cancer Center, Tampa, FL USA; 4 Departments of Radiology & Biomedical Engineering, University of Wisconsin-Madison, Madison, WI USA; 5 Department of Pathology, University Hospitals Cleveland Medical Center, Cleveland, OH USA; 6 Department of Surgery, University Hospitals Cleveland Medical Center, Cleveland, OH USA; 7 Abdominal Imaging and Nuclear Radiology Department, Cleveland Clinic, Cleveland, OH USA; 8 Colorectal Surgery Department, Cleveland Clinic, Cleveland, OH USA; 9 Hematology and Medical Oncology Department, Cleveland Clinic, Cleveland, OH USA; 10 Earle A. Chiles Research Institute, Robert W. Franz Cancer Center, Providence Portland Medical Center, Portland, OR USA; 11 Department of Biomedical Engineering, Emory University, Atlanta, GA USA; 12 Atlanta Veterans Administration Medical Center, Atlanta, GA USA; 13 William S. Middleton Memorial Veterans Affairs Healthcare, Madison, WI USA; 14 Louis Stokes Cleveland Veterans Affairs Medical Center, Cleveland, OH USA

**Keywords:** Cancer therapy, Prognostic markers, Mathematics and computing

## Abstract

With advances in neoadjuvant therapies for rectal cancer, accurately evaluating tumor regression and response is increasingly critical for enabling personalized follow-up, including non-operative management. Given the lack of reliable assessment methods, there is an opportunity to develop computerized image-based markers for identifying early responders. We present a novel radiomic signature, the STructural mOdeling magnituDE and Orientation (StODeO) descriptor for quantifying image-based surrogates of tumor shrinkage within and around the rectum via routine MRI scans. StODeO descriptors measure both the magnitude (how much) and direction (inward/outward) of structural displacements in the diseased rectal wall, with respect to a newly constructed, first-of-its-kind image-based reference atlas of the non-diseased rectum. Combined with traditional radiomic texture features, StODeO showed superior performance in distinguishing pathologic response groups of rectal cancer patients post-chemoradiation (hold-out validation AUC 0.77) as well as segregating composite response groups in a clinical trial of immune checkpoint inhibitors (external validation accuracy 0.71; NCT02688712). The integrated StODeO-texture signature demonstrated robustness across annotation sources and imaging variations, intuitive modeling of therapy-associated structural changes, and statistical associations with tumor-immune biology, including macrophages and CD8+ T-cells from multiplexed biopsy analysis. StODeO offers a novel image-based surrogate of tissue displacements from treatment effects and residual disease after neoadjuvant therapy in rectal cancers.

## Introduction

Colorectal cancer (CRC) is the third most common cancer worldwide with incidence rate of 10%, and over 700,000 patients who are diagnosed with tumors localized to the rectum each year^1^. Furthermore, CRC is the second most common cause of cancer-related deaths with a mortality rate of 9.4% worldwide, despite advances in multimodal therapeutic options such as combining neoadjuvant chemoradiation with chemotherapy^2^ or with immunotherapy^3^. In locally advanced rectal cancers, these therapeutic approaches could enable local control as well as significant tumor regression and thus allow patients to be recommended non-operative management^4^. The latter decision requires evaluating gross morphological and appearance changes within and around rectal tumors to determine treatment-related changes in terms of whether tumor extent has spread outward from the rectal wall into the perirectal fat or invaded inward into the lumen^5^. Such morphological changes are clinically evaluated by radiologists in terms of tumor stage on MR imaging, but the latter has demonstrated only 50–60% agreement with pathologic tumor stage^6,7^ in the post-treatment setting. There is thus a need for more accurate image-based markers of tumor stage response on MRI, which could enable new paradigms for patient evaluation and non-operative disease management in rectal cancers^8,9^.

To address this need, several recent studies have examined computer-extracted quantitative (i.e., *radiomic*) features from MR images for their association with pathologic tumor stage after therapy, in the context of rectal cancers specifically^10–12^. These investigations have largely focused on using radiomic texture descriptors or deep learning strategies to quantify different aspects of tumor appearance on baseline^13–16^ as well as post-therapy^17,18^ MRIs. Interestingly, while these studies have focused on interpreting specific texture or gray-level patterns associated with response, the biological foundation for radiomic measurements remains relatively unexplored. Even more importantly, there may be additional complementary information to be obtained by quantifying morphological and structural changes within and around the rectum due to treatment, beyond using shape descriptors alone^19–22^. For instance, previous studies have found gland shape^23^ as well as gland surface^24^ differences between prostate cancer patients who did and did not suffer biochemical recurrence after definitive therapy, via pre-treatment T2w MRI scans. In glioblastoma multiforme, mass effect-induced deformation heterogeneity in different brain structures on Gadolinium-contrast (Gd-C) T1w-MRI has been found to be associated with patient survival^25,26^. This suggests that detailed quantification of structural modeling changes occurring due to rectal tumor growth or shrinkage could yield key new markers specialized for post-therapy tumor staging of rectal cancers via MRI.

In this work, we develop and investigate a STructural mOdeling magnituDE and Orientation (StODeO) descriptor as an imaging marker of tumor response to therapy in rectal cancers. StODeO is designed as a radiomic signature based on image-based surrogate measurements of tumor shrinkage or growth within and around the rectum via routine MRI scans, in order to evaluate response to neoadjuvant therapy in rectal cancers. More specifically, we (1) construct a novel atlas representation to provide an image-based reference for rectal structures *without* tumor, (2) utilize this atlas in a multi-stage registration and mapping algorithm to quantify both magnitude (i.e., how much structural displacement has occurred within the rectal wall) as well as orientation (i.e., whether structural displacements have occurred inward or outward from the rectal wall) to construct the StODeO descriptor, (3) evaluate our novel StODeO descriptor in conjunction with traditional radiomic descriptors to evaluate response to standard neoadjuvant chemoradiation as well as immune checkpoint inhibitors (in a clinical trial cohort), and (4) examine interpretability of radiomic image signatures (both texture and StODeO) through detailed statistical correlative analysis against corresponding tumor and immune biology phenotypes in rectal cancers.

## Results

Out of 424 patients initially identified from three institutions, 182 were included in this study and were segregated into three cohorts *S*_1−3_:

- *S*_1_ comprised 75 patients diagnosed with a different pelvic cancer (other than rectal, median age: 64.4 ± 7.5 years, 100% male), primarily to provide a clear in vivo MRI visualization of the non-diseased rectal wall prior to any treatment. This was utilized to construct a novel atlas-based image representation of the rectum *without* tumor, based on which novel StODeO descriptors were computed. Further details are provided in Supplementary Material.
- *S*_2_ comprised 88 patients retrospectively identified from two different institutions (median age: 57.0 ± 14.2 years, 60.2% male) who had been diagnosed and treated for locally advanced rectal cancer via standard-of-care neoadjuvant chemoradiation followed by proctectomy. These patients were further segregated into distinct discovery and hold-out validation cohorts. Based on pathologic evaluation of the post-treatment surgical specimens, 36% (32/88) of these patients were confirmed as having achieved pathologic downstaging.
- *S*_3_ comprised 19 patients treated at an external institution (median age: 52.4 ± 12.7 years, 89.5% male) for locally advanced rectal cancer within a prospective clinical trial evaluating an experimental neoadjuvant immune checkpoint inhibitor (ICI) therapy prior to standard-of-care chemoradiation (NCT02688712^3^). Patient response was assessed via a composite of pathological complete response in patients who proceeded to surgery, or clinical complete response maintained at 1 year after last therapy in patients with non-operative management; with 36.8% (7/19) assessed as achieving response.

### Performance of novel StODeO and traditional radiomic descriptors in evaluating pathologic response after standard-of-care chemoradiation therapy for rectal cancers

Table 1 lists the top-ranked descriptors comprising StODeO magnitudes (denoted ${{\mathbb{F}}}_{m}$), traditional radiomic textures (denoted ${{\mathbb{F}}}_{t}$), as well as the combination of the two (denoted ${{\mathbb{F}}}_{mt}$); sorted based on frequency of selection together with their *p*-values from Wilcoxon ranksum testing between response-based patient groups. ${{\mathbb{F}}}_{mt}$ can be seen to comprise two descriptors from ${{\mathbb{F}}}_{m}$ as well as four descriptors from ${{\mathbb{F}}}_{t}$. Note that StODeO orientations (denoted ${{\mathbb{F}}}_{o}$) were only ever considered in combination with ${{\mathbb{F}}}_{m}$ or ${{\mathbb{F}}}_{mt}$, and thus have not been listed for brevity.

**Table 1:** Top-ranked descriptors comprising each of {${{\mathbb{F}}}_{m}$}, {${{\mathbb{F}}}_{t}$}, and {${{\mathbb{F}}}_{mt}$}, together with their unadjusted *p* values from Wilcoxon ranksum testing between patient response groups

Rank	${{\mathbb{F}}}_{m}$	${{\mathbb{F}}}_{t}$	${{\mathbb{F}}}_{mt}$
1	Median—Magnitude Def. (*p* = 0.00005)	Skewness—Gabor XY (*p* = 0.444)	Skewness—Gabor XY (*p* = 0.444)
2	Standard Dev.—Magnitude Def. (*p* = 0.002)	Skewness—CoLlAGe info1 ws = 5 (*p* = 0.0005)	Median—Magnitude Def. (*p* = 0.00005)
3	Skewness—Magnitude Def. (*p* = 0.106)	Variance—CoLlAGe idm ws = 5 (*p* = 0.075)	Skewness—Gradient Sobel YX (*p* = 0.004)
4	Kurtosis—Magnitude Def. (*p* = 0.106)	Skewness—Gradient Sobel YX (*p* = 0.004)	Variance—CoLlAGe idm ws=5 (*p* = 0.075)
5			Standard Dev.—Magnitude Def. (*p* = 0.002)
6			Skewness—CoLlAGe info1 ws=5 (*p* = 0.0005)

Features are ranked based on selection frequency across 50 runs of three-fold cross-validation in the discovery cohort.

**Fig. 1:**
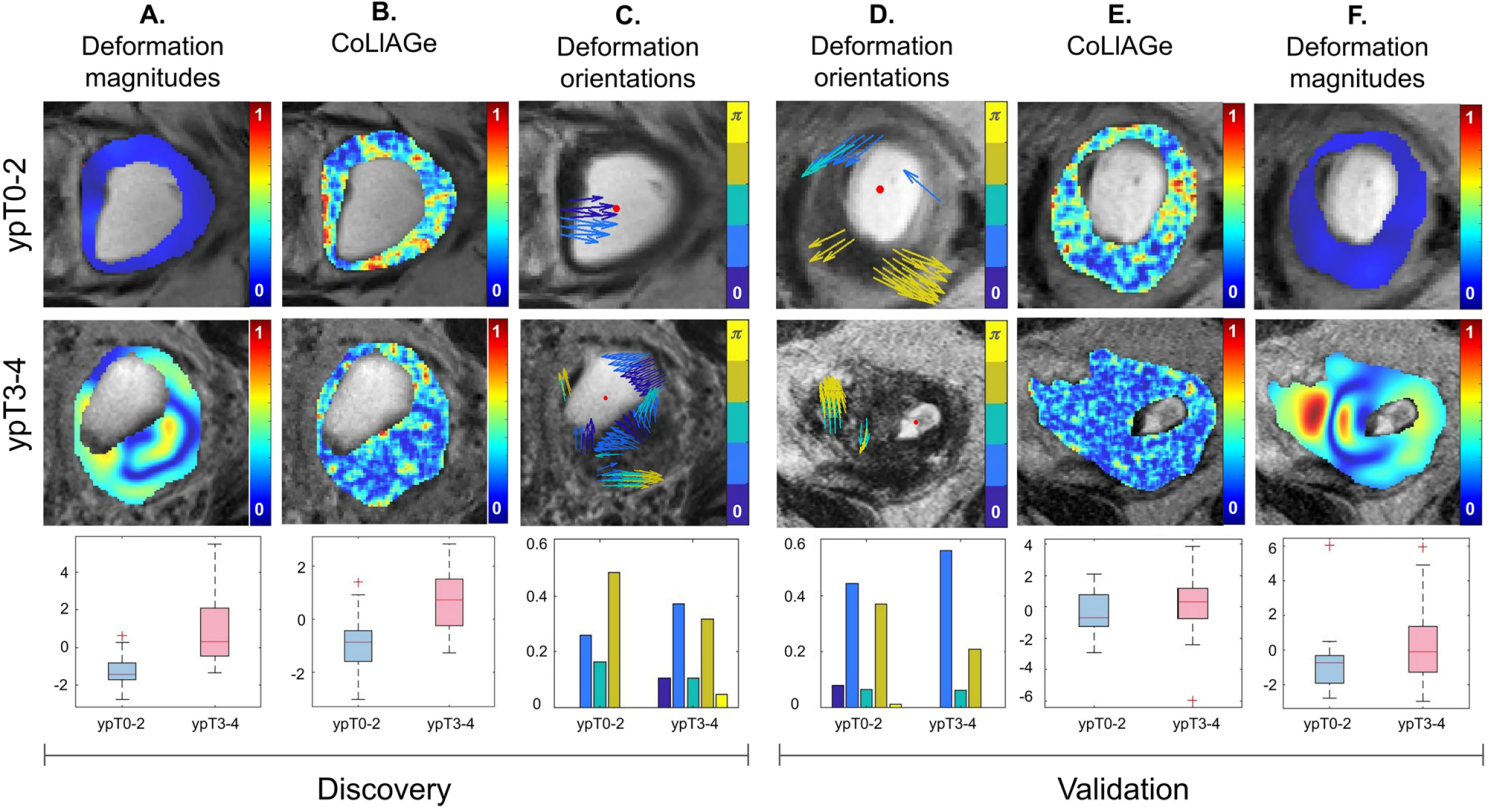
Representative visualizations for StODeO and texture radiomic descriptors between ypT0-2 (top row) and ypT3-4 (bottom row) rectal cancer patients after therapy across discovery (left half) and validation (right half) cohorts. **A**, **F** Heatmaps for structural modeling magnitude within the rectal wall on MRI; red corresponds to significant structural displacements and blue to minimal displacements with respect to the atlas. **B**, **E** Heatmaps for Variance—CoLlAGe idm ws = 5, where red corresponds to over-expression and blue to under-expression. **C**, **D** Orientation maps localized to regions of most significant deformation, where blue arrows indicates deformation vectors were oriented inward to the lumen and yellow arrows indicate vectors oriented outward from the rectal wall. Distinctive differences between patient groups are reflected in corresponding boxplots (for structural modeling magnitude and texture heterogeneity) and histograms (for deformation orientation) in the bottom row, for both cohorts.

Figure 1 depicts representative heatmaps for descriptors from ${{\mathbb{F}}}_{m}$ (StODeO magnitude), ${{\mathbb{F}}}_{t}$ (texture variance—CoLlAGe idm ws = 5), as well as ${{\mathbb{F}}}_{o}$ (StODeO orientation); for low (top row) and high (middle row) pathologic stage rectal cancer patients in each of the discovery and validation cohorts. Patients with ypT0-2 tumors are seen to exhibit significantly lower and less variable structural wall displacements (homogeneous bluish appearance of magnitude heatmap), under-expression of texture heterogeneity, as well as pronounced deformations which are oriented inward or along the wall (arrows pointed inward or along the wall). By comparison, ypT3-4 tumors exhibit variable structural modeling changes (cyanish-red appearance of magnitude heatmap), over-expression of texture heterogeneity, as well as pronounced deformations oriented both inward and outward from the rectal wall.

As summarized in Table 2, the StODeO descriptor (${{\mathbb{F}}}_{mo}$, both magnitude and orientation) yielded significantly improved linear discriminant (LDA) classifier performance for distinguishing patient response groups in the discovery cohort compared to ${{\mathbb{F}}}_{t}$, though both feature sets yield comparable classifier performance in the validation cohort. Combining StODeO with radiomic texture descriptors (denoted ${{\mathbb{F}}}_{mto}$) resulted in the best overall LDA classifier performance with a discovery AUC of 0.86 ± 0.04 and hold-out validation AUC of 0.77 (in 2D). At the optimized threshold in the validation cohort, this corresponds the best overall performance trade-off in identifying ypT0-2 patients after therapy (80% sensitivity, 67% specificity, 52% precision, 63% f-score). Similar trends in classifier performance between ${{\mathbb{F}}}_{mo}$, ${{\mathbb{F}}}_{t}$, and ${{\mathbb{F}}}_{mto}$, can be observed when comparing trends between 2D and 3D descriptors († symbol in Table 2), though overall classifier performance in the former case is significantly higher in terms of AUC, sensitivity, specificity, and precision. Model trends for QDA and RF classifiers are summarized in Supplementary Tables S3 and S4 respectively, which demonstrate similar trends in performance to the LDA classifier. When comparing performance of different 3D feature sets (Supplementary Table S5), performance for ${{\mathbb{F}}}_{mto}$ within the 5-section subvolume was significantly improved compared to utilizing the entire rectal volume. Supplementary Table S6 summarizes classification performance using cTNM variables and rectal wall volume, both of which achieved significantly lower performance than ${{\mathbb{F}}}_{mto}$ in distinguishing patient response groups across both discovery and validation cohorts.

**Table 2:** Classifier performance comparing ${{\mathbb{F}}}_{mo}$, ${{\mathbb{F}}}_{t}$, and ${{\mathbb{F}}}_{mto}$ in both 2D and 3D; via an *LDA* model

	Feat set	${{\mathbb{F}}}_{mo}$ (*n* = 9)		${{\mathbb{F}}}_{t}$ (*n* = 4)		${{\mathbb{F}}}_{mto}$ (*n* = 11)	
	Cohort	Discov.	Valid.	Discov.	Valid.	Discov.	Valid.
2D	AUC	0.79 (0.04)*	0.70	0.66 (0.08)*	0.65	0.86 (0.04)	**0.77**
	Acc	0.78 (0.03)*	0.73	0.69 (0.05)*	0.56	0.83 (0.04)	**0.71**
	Sens	0.7 (0.1)*	0.40	0.5 (0.2)*	0.87	0.87 (0.1)	**0.80**
	Spec	0.85 (0.07)*	0.88	0.83 (0.14)	0.42	0.79 (0.09)	**0.67**
	F-score	0.73 (0.04)*	0.48	0.55 (0.15)*	0.55	0.81 (0.05)	**0.63**
	Prec	0.78 (0.08)	0.60	0.74 (0.14)	0.41	0.76 (0.06)	**0.52**
3D	AUC	0.79 (0.04)*	0.66	0.64 (0.09)*	0.66	0.84 (0.04)^†^	0.66
	Acc	0.8 (0.04)*,^†^	0.67	0.69 (0.05)*	0.60	0.83 (0.03)	0.65
	Sens	0.79 (0.06)^†^	0.47	0.44 (0.22)*,^†^	0.67	0.8 (0.08)^†^	0.53
	Spec	0.81 (0.05)*,^†^	0.76	0.87 (0.11)^†^	0.58	0.85 (0.05)^†^	0.70
	F-score	0.77 (0.04)^†^	0.47	0.52 (0.17)*,^†^	0.51	0.8 (0.04)	0.48
	Prec	0.75 (0.05)*,^†^	0.47	0.77 (0.19)^†^	0.42	0.8 (0.05)^†^	0.44

Mean (standard deviation) of each performance measure are reported across 50 runs of three-fold cross validation in the discovery cohort and for hold-out evaluation in the validation cohort. Top-performing strategy across all feature sets and comparison is bolded.

*Feat set* feature set (number of features), *AUC* area under the ROC curve, *Acc* accuracy, *Sens* sensitivity, *Spec* specificity, *Prec* precision.

*Indicates statistical significance based on *p* value ≤0.05 w.r.t. ${{\mathbb{F}}}_{mto}$.

^†^Indicates statistical significance between corresponding 2D vs. 3D feature sets, based on *p* value ≤0.02.

### Evaluating performance and biological basis for combined StODeO and traditional radiomic descriptors in external clinical trial of ICI therapy in rectal cancers

**Fig. 2:**
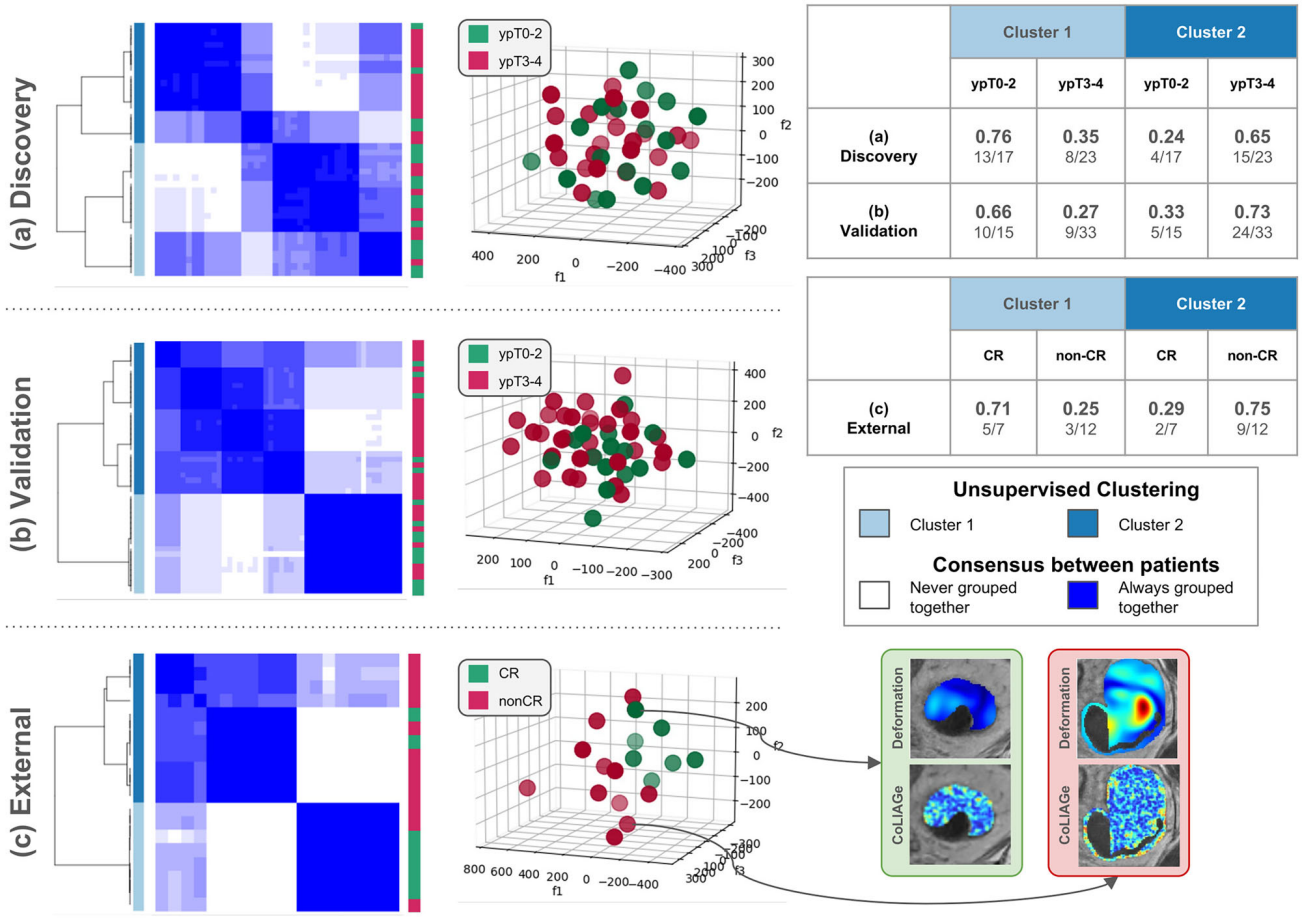
Consensus clustering heatmaps and scatter plots via t-SNE projection of StODeO + texture radiomic descriptors. Rows correspond to different cohorts: **a** discovery, **b** validation (both from S2), **c** external (from S3). Left column: consensus clustering heatmaps where deeper blue shading indicates higher frequency with which a pair of patients was clustered together, together with original response groupings depicted via red-green colorbar alongside. Middle column: 3D scatter plots obtained via t-SNE for **a**, **b** ypT0-2 tumors (green) vs. ypT3-4 tumors (red), and **c** clinical response (green) vs. non-clinical response (red). Right column (upper): unsupervised clustering accuracy for each t-SNE projection showing ${{\mathbb{F}}}_{mto}$ enables accurate segregation of response groupings in all three cohorts. Right column (bottom): heatmaps for StODeO magnitudes and radiomic textures in external cohort S3.

Comparing the 3D scatter plots and unsupervised clustering heatmaps of t-SNE projections corresponding to ${{\mathbb{F}}}_{mto}$ within each of the discovery, validation, and external cohorts in Fig. 2 illustrate that the combination of StODeO magnitudes and radiomic texture descriptors is able to accurately segregate response groupings in all three cohorts. In terms of overlap accuracy of the unsupervised clustering, Cluster 1 in each plot was found to correctly identify 76%, 66%, and 71% of patients who achieved response (ypT0-2 in discovery and validation, composite response in external). This can be further confirmed via the unsupervised clustering heatmap for each projection, which demonstrates that ${{\mathbb{F}}}_{mto}$ accurately identifies a majority of these patients despite the relative class imbalance. Representative heatmaps for StODeO magnitude and radiomic texture in the bottom right of Fig. 2 also depict similar trends to heatmaps shown in Fig. 1.

**Fig. 3:**
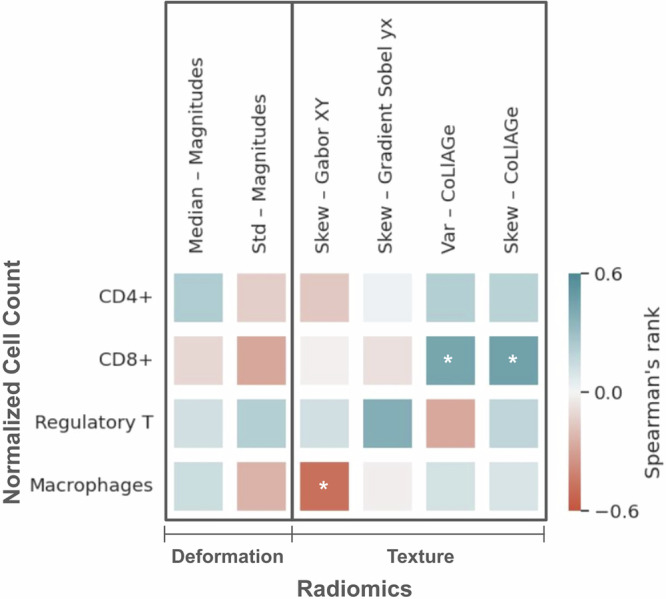
Cell map of Spearman’s rank correlation coefficients for normalized cell count (*Y*-axis) vs. top-ranked StODeO magnitude and radiomic texture descriptors (*X*-axis) where red = negative correlation, blue = positive correlation. White asterisks (*) indicate potential correlations after false discovery rate correction.

Figure 3 visualizes a cell map of correlations between top-ranked StODeO magnitude and radiomic texture descriptors comprising ${{\mathbb{F}}}_{mto}$ and normalized cell counts of immune infiltrate cell types (determined via multiplex immunohistology of biopsies obtained after experimental ICI therapy in *S*_3_). After false discovery rate correction, CD8+ and macrophage cell counts were found exhibit the highest correlations with specific radiomic texture descriptors, and only weak correlation with StODeO magnitude descriptors.

### Robustness and reproducibility of StODeO magnitude and traditional radiomic descriptors

Table 3 summarizes the results of Wilcoxon ranksum testing of descriptors comprising ${{\mathbb{F}}}_{mto}$ when evaluated with respect to patient sex (male vs. female), magnetic field strength (1.5 vs. 3T), annotation scheme (manual vs. automated), institution (UHCMC vs. CCF), or scanner manufacturer (Siemens vs. Philips). No significant differences can be observed in any of StODeO magnitude and radiomic texture descriptors, with respect to any of these variables (all *p* > 0.004, Bonferroni-corrected threshold). Robustness of StODeO descriptors to acquisition-related differences (magnetic field strength, scanner manufacturers) can be additionally verified by their instability scores falling within a relatively low range of values (0.04–0.12), summarized in Table S7.

**Table 3:** Evaluating descriptor robustness and reproducibility within ${{\mathbb{F}}}_{mto}$ between groups defined based on patient sex (Sex), magnetic field strength (Mag.F), manual vs. automated annotation (Annot.), institution where the image was acquired (Instit.), as well as scanner used for image acquisition (Scanner)

		Skewness Gabor XY median (IQR)	Median magnitude deformation median (IQR)	Skewness gradient Sobel YX median (IQR)	Variance CoLlAGe idm ws = 5 median (IQR)	Std dev magnitude deformation median (IQR)	Skewness CoLlAGe info1 ws = 5 median (IQR)
Sex	Male (53)	−0.46 (−1.59 to 0.81)	−0.52 (−1.41 to 0.66)	−0.18 (−0.85 to 0.96)	−0.42 (−1.19 to 0.52)	−0.26 (−1.17 to 0.86)	−0.19 (−1.21 to 0.99)
	Female (35)	0.18 (−1.16 to 1.26)	−0.54 (−1.43 to 0.76)	0.1 (−1.2 to 0.94)	0.05 (−0.87 to 1.13)	−0.27 (−1.42 to 1.75)	−0.21 (−0.82 to 1.14)
	*p* value	*0.335*	*0.785*	*0.785*	*0.207*	*0.986*	*0.658*
Mag.F	1.5T (34)	−0.87 (−1.82 to 1.2)	−0.42 (−1.52 to 0.82)	0.12 (−0.86 to 0.66)	−0.25 (−1.05 to 1.08)	−0.62 (−1.4 to 1.09)	−0.17 (−0.84 to 0.94)
	3T (54)	−0.41 (−1.13 to 0.95)	−0.54 (−1.4 to 0.63)	−0.09 (−0.95 to 1.3)	−0.29 (−1.09 to 0.99)	−0.21 (−1.3 to 0.73)	−0.2 (−1.08 to 1.17)
	*p* value	*0.339*	*0.99*	*0.54*	*0.915*	0.8	*0.867*
Annot.	Manual (67)	−0.68 (−1.59 to 0.96)	−0.52 (−1.39 to 0.72)	−0.19 (−0.89 to 0.97)	−0.32 (−1 to 1.08)	−0.22 (−1.35 to 0.87)	−0.2 (−1.06 to 1.14)
	UNet (21)	0.02 (−1.02 to 0.93)	−0.54 (−1.6 to 0.62)	0.21 (−1.65 to 0.82)	−0.27 (−1.29 to 0.47)	−0.37 (−1.35 to 1.17)	−0.15 (−0.63 to 1.03)
	*p* value	*0.347*	*0.776*	*0.93*	*0.792*	*0.922*	*0.725*
Instit.	UHCMC (61)	−0.14 (−1 to 0.96)	−0.54 (−1.42 to 0.66)	0 (−0.91 to 0.99)	−0.27 (−1.18 to 0.89)	−0.25 (−1.34 to 0.84)	−0.17 (−0.89 to 1.13)
	CCF (27)	−1.58 (−2.1 to 1.03)	−0.52 (−1.47 to 0.67)	0.14 (−1.01 to 0.68)	−0.36 (−0.89 to 1.08)	−0.59 (−1.33 to 0.97)	−0.2 (−1.08 to 1.1)
	*p* value	*0.04*	*0.758*	*0.914*	*0.835*	*0.849*	*0.745*
Scanner	Siemens (75)	−0.26 (−1.32 to 1.18)	−0.52 (−1.41 to 0.89)	0.17 (−0.85 to 1.01)	−0.23 (−0.98 to 1.08)	−0.26 (−1.33 to 1.07)	−0.2 (−0.99 to 0.93)
	Philips (13)	−0.81 (−1.79 to −0.03)	−0.54 (−1.56 to 0.16)	−0.85 (−1.48 to 0.54)	−0.97 (−1.39 to 0.55)	−0.27 (−1.43 to 0.23)	0.4 (−0.79 to 1.32)
	*p* value	*0.151*	*0.452*	*0.18*	*0.173*	*0.41*	*0.417*

Median and inter-quartile range (IQR) of feature values are provided together with the *p* value from Wilcoxon ranksum testing.

## Discussion

Accurate non-invasive evaluation of treatment response in rectal cancers has substantial implications for personalizing follow up especially with the advent of novel therapies to boost tumor regression, such as combination of standard of care chemoradiation with chemotherapy or with immune checkpoint inhibitors (ICI). In this study, we presented a novel radiomic signature, the STructural mOdeling magnituDE and Orientation (StODeO) descriptor, which captures image-based surrogates of tumor shrinkage after therapy in rectal cancers. In multi-institutional evaluation, we found that StODeO descriptors independently as well as in conjunction with radiomic texture descriptors yielded accurate performance in identifying patients who achieved pathologic downstaging after chemoradiation as well as those who achieved composite clinical response in a trial of ICI therapy. StODeO and radiomic texture descriptors also demonstrated intuitive structural modeling and heterogeneity patterns between patient response groups in rectal cancers, and were found to be associated with specific tumor and immune biology rectal cancer phenotypes.

Developing the StODeO descriptor involved a unique effort to quantify tumor response-related image displacements on MRI in terms of both the magnitude as well as the orientation of structural modeling changes after therapy. This builds off previous efforts to capture subtle tissue deformations due to disease impact on radiographic imaging in brain^26,27^, lung^28^, and prostate^29^. In our study, structural modeling magnitudes were extracted as a surrogate for how significant and variable structural rectal wall deformations were (with respect to the non-diseased rectal atlas), while the directionality of the deformation vector provided a surrogate for tumor shrinkage or growth (inward or outward from the lumen). We found that patients showing poor response (i.e., higher pathologic stage after chemoradiation or incomplete response after ICI therapy) were associated with larger and more variable structural deformations which occurred both inward or outward from the lumen. Such deformation patterns are physiologically intuitive given the known locoregional impact of aggressive non-responsive tumors^30^, which tend to intrude into the lumen or extend into the surrounding perirectal fat. By contrast, patients with pathologically regressed tumors after chemoradiation or clinical response after ICI therapy were associated with less pronounced deformations that were oriented inward or along the rectal wall. The latter finding likely indicates that rectal structures in patients who most benefited from neoadjuvant therapy may have more similarities in appearance to non-diseased rectal anatomy (as represented by the atlas) as a result of tumor extent shrinking to within the wall. The intuition of deformation patterns via StODeO descriptors also resonate with previous clinical observations of the associated anatomy after therapy in rectal cancers^31^. We also found that routine clinical evaluation (cTNM staging, rectal wall volume) performed significantly worse than StODeO descriptors in identifying pathologic regression after treatment, suggesting the latter could help overcome known issues with clinical staging of rectal cancers^6,7^.

Prior related radiomics approaches for assessment of treatment response in rectal cancers have largely been focused on pre-CRT MRIs alone^16,32–34^ or quantifying texture changes between pre-, mid- and post-treatment MRIs^35–37^. There have been some recent efforts in developing and applying radiomics approaches specifically to post-CRT MRIs alone in rectal cancers^22,38,39^, despite challenges resulting from the more obfuscated and noisy appearance of MRIs after treatment. Notably, previous work has either leveraged data from a single institution^38,40^ or evaluated the combination of traditional radiomics features (texture, shape, edge) in a multi-institutional setting^22,39^. Our current study not only utilized a multi-institutional cohort with hold-out validation but also included unique evaluation of our novel StODeO descriptors in a prospectively accrued clinical trial of ICI therapy in rectal cancers. In both supervised and unsupervised evaluation, integrating StODeO descriptors with traditional radiomic features demonstrated significantly improved performance over either feature set individually in terms of sensitivity, specificity, and accuracy for identifying pathologically regressed or clinically responding rectal cancer patients; suggesting complementarity to the information being captured by either approach. For instance, while StODeO descriptors have been designed to capture “global” magnitudes and orientations of structural modeling changes and displacements in the rectal wall, radiomic descriptors such as CoLlAGe^41^ are intended to quantify more subtle, “local” textural variations of the rectal wall appearance. Interestingly, our radiomic descriptors yielded optimal classification performance when considering the largest section of the wall suspicious for tumor (whether in 2D or 3D). This location may thus represent the most informative sub-region for analysis of structural modeling changes occurring as a result of treatment, suggesting that incorporating additional 2D sections with predominantly healthy tissue did not accurately reflect structural or textural differences of interest in our analysis.

When evaluating associations between radiomic signatures from our study with corresponding tumor and immune biology phenotypes, exploratory analysis revealed potential associations between traditional texture descriptors and CD8+ and macrophage cell counts within biopsy specimens acquired after treatment. Specifically, we found that elevated expressions of texture features capturing chaotic organization of local intensity gradients and heterogeneity were correlated with the presence of cell types which have been shown to play a crucial role in the adaptive immune response to cancer^42–44^. These findings resonate with previously reported associations between radiomic features and tumor biology in other cancers such as breast^45–47^, prostate^48^, lung^49^, glioblastoma^50^, or soft-tissue sarcoma^51^ We however did not determine significant correlations between StODeO descriptors and immune infiltrate cell counts. Upon reflection, this may make intuitive sense when considering the complexity of the biological processes involved in therapy response. We posit that traditional texture descriptors reflect phenotypes within the tumor region specifically and thus demonstrate closer associations immune cell infiltrates (even accounting for sampling issues with the biopsies). By contrast, StODeO descriptors quantify structural changes along the entire rectal wall (including regions surrounding or distant from the tumor), which could explain their weaker association with specific tumor biology as well as confirm their complementarity to traditional radiomic textures.

Medical imaging atlases have been shown to be a useful tool for capturing structural and anatomical variability of organs across a population in terms of a unified reference or canonical representation. Atlases have been constructed for studying structural and appearance trends in healthy subjects while accounting for inter-subject variability in both neurological and cardiac settings^52,53^, as well as to characterize disease appearance for multiple diseases and cancers^54,55^. To our knowledge, our work represents a first attempt at constructing a structural rectal atlas, providing a canonical representation to describe healthy anatomy of the rectum and the lumen. Developing our rectal atlas required accounting for the “hollow tube” structure of the rectum, necessitating a multi-stage process incorporating rigid, affine, as well as deformable registration. We leveraged existing pelvic MRIs from other cancers to construct our atlas, thereby ensuring an accurate reference for non-diseased rectums (which are often included in the field of view for prostate or endometrial cancer scans). Quantitative and qualitative evaluation further demonstrated our atlas to be a relatively robust and accurate structural representation of the rectum and the lumen, suggesting our framework and representation could be crucial for future studies in this domain.

We do acknowledge some limitations to our study. Our non-diseased rectal atlas used pelvic MRIs from a male population alone, as they were from prostate cancer patients. However, we minimized the potential impact of anatomic and appearance differences in the pelvis between males and females by constructing a *structural* atlas of the rectal wall and lumen alone (rather than focusing intensity differences). We also performed a comprehensive evaluation to confirm the reproducibility of top-ranked StODeO and radiomic texture descriptors between sex-based patient groups (in addition to several other variables). Although we utilized retrospectively accrued data from multiple institutions, our overall cohort size may be considered limited. While this was partially driven by stringent inclusion criteria where only axial T2-weighted MRI scans were considered (to minimize the impact of differences in rectal structures between axial and coronal views), we nevertheless robustly evaluated our models on prospectively accrued data from a novel clinical trial of ICI therapy in rectal cancers. Notably, despite the heterogeneity across our validation cohorts, our model demonstrated consistent performance. There may be concerns regarding the reproducibility of the StODeO to annotation differences resulting from inter-observer variability. To account for this, we evaluated the StODeO descriptors using annotations generated manually as well as automatically (via a U-net), and found no significant differences; suggesting this may not be a significant concern.

In conclusion, we presented a new STructural mOdeling magnituDE and Orientation (StODeO) radiomic descriptor designed to capture both the degree and directionality of image-based structural modeling changes on MRI, as a surrogate of tumor shrinkage or growth after therapy. We found that integrating StODeO descriptors with radiomic texture descriptors yielded the best overall performance in accurately and intuitively evaluating response to multiple types of neoadjuvant therapy in rectal cancers. Future work will include utilizing our atlas and deformation framework to examine population differences in rectal cancers as well as larger scale validation of our signature in order to confirm its ability to inform selection of non-operative management vs. surgery in these patients.

## Methods

This HIPAA-compliant, retrospective study was approved by institutional review boards (IRBs) at three institutions, University Hospitals Cleveland Medical Center (UHCMC, #07-16-40), Cleveland Clinic Foundation (CCF, #18-427), and the Providence Cancer Institute (PCC); with a waiver for requirement of informed consent as de-identified patient data was utilized.

### Study population

This multi-institutional study included patients diagnosed with multiple pelvic cancers, as summarized below:

- *S*_1_: a total of 75 patients who had been diagnosed with prostate cancer and had undergone an axial pelvic MRI scan at diagnosis (prior to any treatment) between September 2014 and April 2016 were curated from UHCMC. Inclusion criteria were: (1) no endorectal coil used during axial MRI acquisition, (2) clear in vivo visualization of the rectal wall with no acquisition or motion artifacts, (3) no tumor invasion into the rectum. Study radiologists (10+ years of experience) reviewed each scan to ensure there was neither tumor invasion into the rectum (i.e., a non-diseased rectum) nor significant displacement of the rectum due to prostate enlargement.
- *S*_2_: a total of 311 patients diagnosed with rectal cancer between August 2007 and June 2019 were initially identified from UHCMC (Institution 1) and CCF (Institution 2). Inclusion criteria were: (1) biopsy-proven locally advanced rectal adenocarcinomas, (2) neoadjuvant chemoradiation prior to total mesorectal excision, (3) availability of axial T2w MRIs after chemoradiation and prior to total mesorectal excision, and (4) availability of pathology reports based on excised specimens. Patients with no therapy (*n* = 13), short-course therapy (*n* = 18), no availability of axial post-therapy T2w MRI (*n* = 156), coronal view (*n* = 22), missing pathologic or clinical information on report (*n* = 9), or sigmoid rectal cancers (*n* = 5) were excluded. Across both institutions, 88 patients were curated as having met all criteria. All included patients had undergone long-course chemoradiation involving 45 to 50.4 Gy in 25 to 28 fractions over 5 to 6 weeks, with concomitant chemotherapy consisting of oral Capecitabine 825 to 850 mg/m^2^ (BID) on days of radiation therapy. Dosages and durations varied slightly at each institution (though the regimen was the same), and after which an MRI scan had been acquired for post-therapy evaluation. All patients underwent a proctectomy at a median of 28 days (range: 6–83 days) after chemoradiation. Study pathologists had assessed and recorded tumor-node-metastasis (ypT-N-M) staging of the excised specimens according to AJCC guidelines^56^ into clinical reports for each patient, which were curated during retrospective data collection. This pathologic stage assessment of post-surgical specimens was used as the ground-truth reference such that tumor down-staging was defined as ypT0-2 (i.e., a lower pathologic stage than the original clinical stage, also implying minimal or dying tumor within the rectal wall).
- *S*_3_: a total of 38 participants diagnosed with rectal cancer between October 2016 and August 2020 were enrolled at PCC (Institution 3) as part of a prospective clinical trial of immune checkpoint inhibitors (ICI) prior to chemoradiation (NCT02688712). Inclusion criteria for our analysis were: (1) availability of axial T2w MRI acquired 15 days after ICI therapy, (2) consistent lumen structure visible on MRI, (3) availability of clinical reports, including clinical response assessment. Study radiologists and biomedical engineers confirmed all criteria. A total of 19 patients were found to meet these inclusion criteria. All study participants had completed two 14-day courses of oral galunisertib 150 mg twice daily, before and during fluorouracil-based chemoradiation (intravenous fluorouracil 225 mg/m^2^ over 24 h daily 7 days per week during radiotherapy or oral capecitabine 825 mg/m^2^ twice per day 5 days per week during radiotherapy; radiotherapy consisted of 50 ⋅ 4–54 ⋅ 0 Gy in 28–30 fractions). Fifteen days after the ICI course, a pelvic MRI scan had been acquired as well as an intraluminal forceps biopsies of the primary tumor. Biopsies were formalin fixed before tissue processing and paraffin embedding, followed by immunofluorescent staining. Multiplex immunohistology was conducted together with scanning of multiple regions of interest per tissue section, with imaging software-based analysis to obtain cell counts for CD8+ T cells, CD4+ T cells, regulatory T cells, and macrophages. Five to nine weeks after treatment, all patients had undergone response assessment. Patients assessed with a complete clinical response could opt for non-operative management and proceed to modified FOLFOX6 (intravenous leucovorin 400 mg/m^2^ on day 1, intravenous fluorouracil 400 mg/m^2^ on day 1 then 2400 mg/m^2^ over 46 h, and intravenous oxaliplatin 85 mg/m^2^ on day 1 delivered every 2 weeks for eight cycles) or CAPEOX (intravenous oxaliplatin 130 mg/m^2^ on day 1 and oral capecitabine 1000 mg/m^2^ twice daily for 14 days every 3 weeks for four cycles). Patients evaluated as less than complete response underwent surgical resection. The primary endpoint was complete response rate, which was a composite of pathological complete response in patients who proceeded to surgery, or clinical complete response maintained at 1 year after last therapy in patients with non-operative management. Complete details for this study can be found in the associated publication^3^.

### T2w MRI acquisition, post-processing and annotation

Imaging data was acquired as a series of DICOM images saved directly from the scanners, encompassing three different scanner manufacturers and eleven different models across three different institutions, though the range of acquisition parameters was consistent within each institution. Scanner and imaging parameters used at each institution are summarized in Supplementary Table S1.

Based on available clinical, pathology, and radiology reports (as well as any additional imaging planes and sequences), a biomedical engineer in collaboration with an expert radiologist manually annotated the entire rectal wall on each T2w MRI dataset via hand-annotation tool in *3D Slicer*^57^, in *S*_1_, *S*_2_, and *S*_3_. Additionally, for a subset of 21 patients from *S*_2_, the rectal wall was automatically annotated via a previously presented region-specific UNet convolutional neural network architecture^58^. To account for differences in voxel resolution across the institutions, all imaging data and their respective annotations were linearly resampled to the most consistently occurring resolution (0.781 mm × 0.781 mm × 4.0 mm); using *3D Slicer*.

### Computing the STructural mOdeling magnituDE and Orientation (StODeO) descriptor

**Fig. 4:**
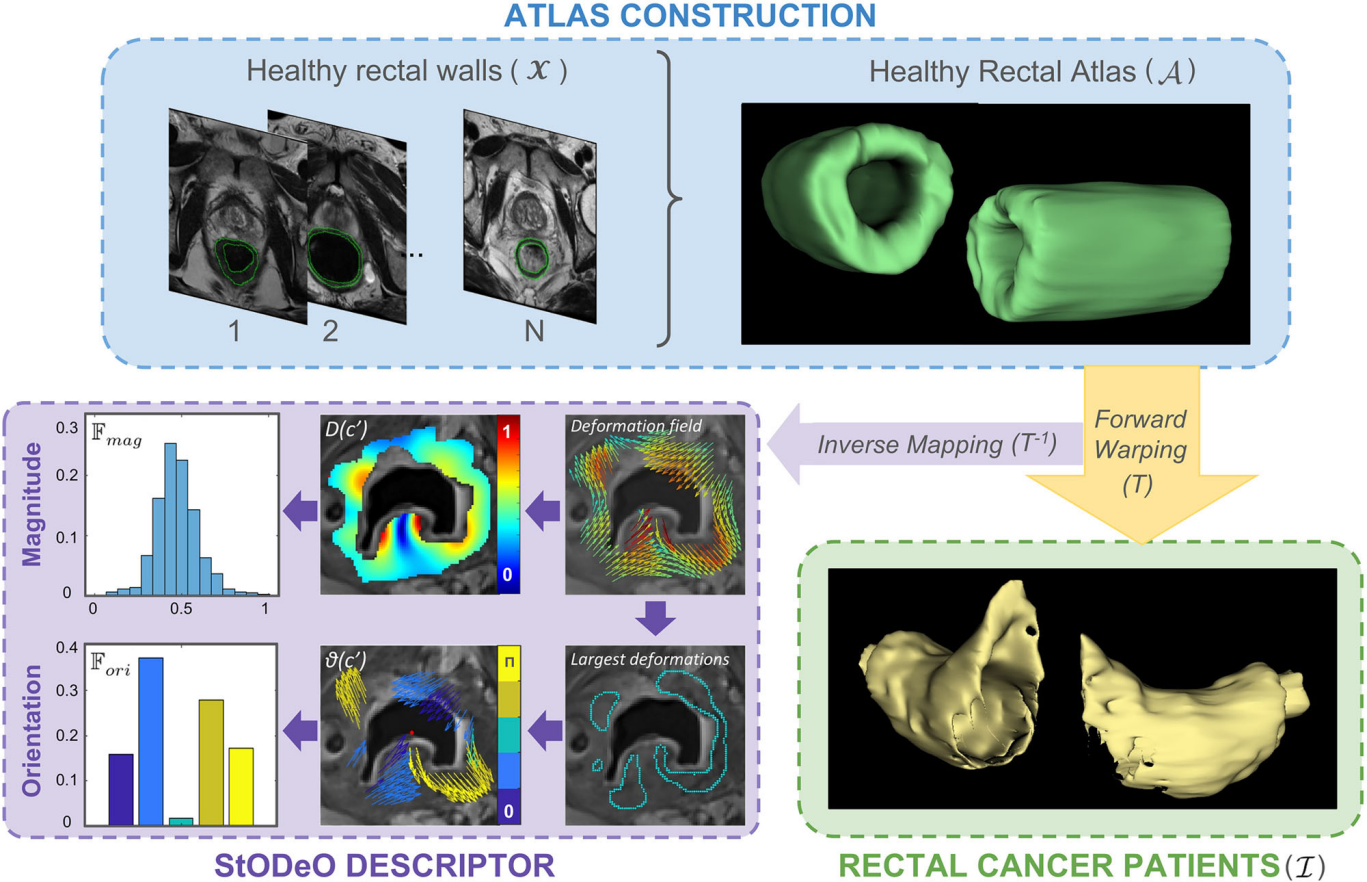
Overall workflow for extracting the StODeO descriptor. Steps include construction of healthy rectal atlas, computing structural modeling changes within the rectal wall in rectal cancer patients (with respect to the atlas), and extracting structural magnitude- and orientation-based features within the rectal wall.

This involves first constructing a structural rectal atlas, computing structural displacements with respect to the non-diseased atlas, based on which the StODeO magnitude and orientation descriptors can be derived. Figure 4 depicts an overview of the steps involved in computing the StODeO descriptor.

We consider a set of *N* MRI scenes depicting healthy rectal anatomy, which are aligned through multiple registration stages into a canonical space to construct the non-diseased rectal atlas, denoted ${\mathcal{A}}$. Further details of rectal atlas construction are provided in Supplementary Material, while notation used in this section is summarized in Supplementary Table S2.

Given a rectal cancer patient MRI scene, denoted ${\mathcal{I}}$, structural modeling changes in the rectal environment are quantified with respect to the non-diseased rectal atlas ${\mathcal{A}}$. First, ${\mathcal{A}}$ is non-rigidly registered to ${\mathcal{I}}$ using a normalized mutual information-based similarity measure within a b-spline registration scheme. This non-rigid alignment is formulated as $\bar{{\mathcal{A}}}=T({\mathcal{A}},{\mathcal{I}})$, where *T* is the forward transformation of the composite voxel-wise deformation field (comprising affine and deformable components) that maps the ROI between the reference (${\mathcal{I}}$) and floating (${\mathcal{A}}$) volumes. This transformation is then inverted to yield *T*^−1^, which is used to map ${\mathcal{I}}$ into the ${\mathcal{A}}$ space, to yield ${{\mathcal{I}}}^{{\prime} }$. This two-stage mapping process is designed to compute structural modeling changes within ${\mathcal{I}}$ with respect to ${\mathcal{A}}$ at every voxel location, thus ensuring that all StODeO descriptors are defined with respect to the atlas space.

Robust atlas construction and computation of the StODeO descriptors were ensured by implementing the following strategies during co-registration: (1) initialization by centering and cropping ${\mathcal{I}}$ with respect to the rectum to have the same size as ${\mathcal{A}}$, (2) starting each registration of ${\mathcal{A}}$ and ${\mathcal{I}}$ with a rigid transform (global displacements and rotations) followed by non-rigid transformation (local transforms and elastic deformations), (3) utilizing the normalized mutual information to drive registration as it is more robust to intensity variations or noise.

Structural modeling changes are then quantified for every transformed cancer patient scene ${{\mathcal{I}}}^{{\prime} }$. Considering $({c}_{x}^{{\prime} },{c}_{y}^{{\prime} },{c}_{z}^{{\prime} })$ as the new voxel positions of every voxel ${c}^{{\prime} }\in {C}^{{\prime} }$ within ${{\mathcal{I}}}^{{\prime} }$, the corresponding displacement vector with respect to the original voxel locations *c* ∈ *C* is given as [*δx*, *δy*, *δz*] where vector $({c}_{x}^{{\prime} },{c}_{y}^{{\prime} },{c}_{z}^{{\prime} })=({c}_{x},{c}_{y},{c}_{z})+(\delta x,\delta y,\delta z)$. Based on this displacement vector, the structural modeling magnitude and orientation which comprise the StODeO descriptor are computed as follows:

- Magnitudes are first calculated at every voxel location ${c}^{{\prime} }\in {C}^{{\prime} }$ within the patient scene ${{\mathcal{I}}}^{{\prime} }$ based on the Euclidean norm of the deformation vector:
  1 $$D({c}^{{\prime}})=\sqrt{{(\delta x)}^{2}+{(\delta y)}^{2}+{(\delta z)}^{2}}.$$
  To quantify how significant the structural displacements are with respect to the non-diseased atlas, first order statistics (i.e., median, standard deviation, skewness, and kurtosis) of *D* are calculated across all voxel locations within ${{\mathcal{I}}}^{{\prime} }$, to yield the magnitude descriptor ${{\mathbb{F}}}_{m}$.
- Orientations are only calculated at those voxel locations within ${{\mathcal{I}}}^{{\prime} }$ that exhibit the largest magnitude deformations, with the assumption that these locations correspond to the most relevant response-related disruptions within the ROI. These regions can be identified by spatially clustering the deformation magnitude values $D({c}^{{\prime} }),\forall {c}^{{\prime} }\in {C}^{{\prime} }$ via Markov random field (MRF)-based clustering^59^. The MRF algorithm labels image pixels based on the description of their statistical and contextual information (spatial dependence between pixels and their labels), isolating rectal wall regions with the most significant structural modeling changes. MRF clustering parameters were empirically set as: number of classes (6), number of iterations (8), and the potential to define the energy of label field (0.5). Intuitively, vectors oriented outward with respect to the ROI centroid likely represent tumor growth while vectors oriented inward likely correspond to tumor shrinkage. Considering ${v}^{{\prime} }$ as the deformation vector associated with voxel location ${c}^{{\prime} }$, its deformation angle can be computed with respect to the ROI centroid based on a geometric *arccos* transform as shown in Supplementary Fig. S3, as:
  2 $$\theta ({c}^{{\prime} })=\arccos \left(\frac{{v}^{{\prime} }\cdot {v}^{o}}{\parallel {v}^{{\prime} }\parallel \parallel {v}^{o}\parallel }\right),$$
  where *v*^*o*^ is the vector defined as $\vec{{c}^{{\prime} }{c}^{o}}$ between the voxel location ${c}^{{\prime} }$ and the lumen centroid *c*^*o*^. For instance, *θ* ∈ [0–20°] indicates deformation vectors oriented toward the centroid (i.e., inward force), while *θ* ∈ [160–180°] corresponds to outward deformation vectors. Orientations in *θ* are quantized into 5 bins to ensure sufficient discretization (0–20°, 20–80°, 80–100°, 100–160°, and 160–180°), to yield the orientation descriptor, ${{\mathbb{F}}}_{o}$.

### Extracting StODeO and traditional radiomic descriptors

For every scan in *S*_2_ and *S*_3_, the StODeO descriptors ${{\mathbb{F}}}_{m}$ (4 × 1 vector) and ${{\mathbb{F}}}_{o}$ (5 × 1 vector) were calculated as described in the previous section. Radiomic texture features were also extracted for all scans to characterize the appearance of the rectal wall, yielding ${{\mathbb{F}}}_{t}$ (764 × 1 vector). All radiomic descriptors were extracted from a single 2D section in each MRI dataset which comprised the largest area of rectal wall suspicious for tumor, assuming that this region was most likely to exhibit signatures related therapy response. For comparison purposes, StODeO and texture features were also extracted in 3D from a sub-volume of 5 consecutive largest 2D sections comprising the slice with largest wall area suspicious for tumor, as well as from the entire rectal volume as a whole. All feature calculations were implemented in *MATLAB* (Mathworks, MA).

### Experimental evaluation

To ensure robustness, *S*_2_ was segregated into: (1) a discovery cohort comprising 40 studies from UHCMC, and (2) a hold-out validation cohort comprising 48 studies: 21 patients from UHCMC, and all 27 patients from CCF. Separately, *S*_3_ was utilized as an external validation cohort. Quantitative and qualitative evaluation to confirm accurate construction of the rectal atlas ${\mathcal{A}}$ via *S*_1_ have been described in Supplementary Material.

Supervised machine learning evaluation involved first implementing feature selection based on the minimum redundancy maximum relevance^60^ (mRMR) scheme, to identify the most discriminant descriptors within each of ${{\mathbb{F}}}_{m}$ and ${{\mathbb{F}}}_{t}$, yielding three feature sets: (1) ${{\mathbb{F}}}_{mo}$=[${{\mathbb{F}}}_{m},{{\mathbb{F}}}_{o}$], (2) ${{\mathbb{F}}}_{t}$, (3) ${{\mathbb{F}}}_{mto}$=[${{\mathbb{F}}}_{m},{{\mathbb{F}}}_{t},{{\mathbb{F}}}_{o}$]. Classification performance for each feature set was evaluated via a binary linear discriminant analysis (LDA) classifier, that was trained to distinguish between low (ypT0-2) and high (ypT3–4) pathologic tumor stages as a marker of response to therapy. The LDA classifier was specifically chosen to enable fairer comparisons between singleton and non-singleton descriptors by projecting each into a lower dimensional space^61^. For comparison purposes, feature sets were additionally evaluated via a quadratic discriminant analysis (QDA) classifier and a random forest (RF) classifier^62^. In these experiments, feature selection and classifier construction utilized the discovery cohort alone, with 50 runs of 3-fold cross-validation to ensure robustness. Per run, bootstrapped AUC values were computed, for a total of 50 AUCs. These were then compared using a nonparametric Kruskal-Wallis test together with multiple comparison correction of the *p* value to confirm statistical significance. Testing of the optimized classifiers were conducted in hold-out fashion on the validation cohort, with the area-under-the-receiver-operator-characteristic curve (AUC), accuracy, sensitivity, specificity, f-score, and precision used as measures of performance. All analyses were conducted using 2D and 3D feature sets, separately. As a baseline for performance via clinical evaluation, LDA models were additionally trained using (1) clinical TNM (cTNM) staging available from patient records, and (2) volume of the rectal wall (computed based off annotated regions). Non-parametric statistical testing was performed to determine (1) significant differences in performance between different feature sets, (2) differences in performance between 2D or 3D feature sets, and (3) radiomic feature robustness and reproducibility between subgroups based on magnetic field, sex, annotation, institution, and scanner differences. A secondary analysis of radiomic feature robustness against imaging variations specifically was conducted via the instability score^63^ (scale: 0 to 1, lower IS values imply that a feature remains largely unaffected by acquisition-related differences). Bonferroni correction was implemented to account for multiple comparisons evaluated in each case.

Unsupervised evaluation of the integrated StODeO-texture radiomic signature involved projecting ${{\mathbb{F}}}_{mto}$ into 3 dimensions via the t-SNE algorithm^64^ (with random initialization, 30 nearest neighbors and 1000 iterations by Euclidean metric) for each of *S*_1−3_. As this tool has been shown to optimally preserve non-linear high-dimensional relationships into lower-dimensional spaces, naturally occurring clusters in the data could be easily visualized via a 3D scatter plot of each t-SNE space. Quantitative evaluation of these clusters was done via consensus clustering of each t-SNE projection using the ConsensusClusterPlus package in R^65^, with 1000 iterations of hierarchical consensus clustering (*k* = 2) by Pearson distance and 80% random patient resampling between runs. Clustering results were visualized using a consensus cluster heatmap where deeper blue shading indicated a higher frequency of clustering together each pair of patients across all runs. Clustering results were also compared against response groupings, to quantify the ability of top features to identify each of the two groups in an unsupervised fashion. To explore the biological basis of the integrated StODeO-texture radiomic signature, correlation coefficients and associated *p* values were computed within *S*_3_ for each pair of variables (radiomic descriptor vs. normalized cell count) using Spearman’s rank correlation test, which is robust to outliers and quantifies monotonicity^66^. Non-relevant correlations were discarded using positive False Discovery Rate (FDR)^67^ for multiple hypothesis testing, identified as those with *FDR* > 0.3. This threshold was defined considering the cohort size and exploratory nature of the analysis.

## Supplementary information

Supplementary Information

## Data Availability

Since the cases from the involved institutions are protected through institutional compliance, the clinical repository of cases can only be shared per specific institutional review board (IRB) requirements. Upon reasonable request, a data sharing agreement can be initiated between the interested parties and the clinical institution following institution-specific guidelines. This is applicable for Cohort S1, S2, S3 from University Hospitals Cleveland Medical Center, Cleveland Clinic Foundation, and Providence Cancer Institute.

## Code availability

All relevant codes are available from the corresponding authors upon reasonable request.
