# Supplementary material for: A novel structural modeling magnitude and orientation radiomic descriptor for evaluating response to neoadjuvant therapy in rectal cancers via MRI
Source: NPJ Precis Oncol. 2025 Jul 1;9:215. doi: 10.1038/s41698-025-01007-3 (PMC12216517; doi:10.1038/s41698-025-01007-3)
Supplement: Supplementary file 1 — Supplementary Information [file 41698_2025_1007_MOESM1_ESM.pdf]

Supplementary Material:

Construction and evaluation of healthy rectal atlas

We consider a set of N MRI scenes depicting the healthy anatomy denoted $\mathcal{X}_i = (C, f)$, where $i \in \{1, ..., N\}$ is a subject scene comprising voxels $c \in C$, which are positioned in a 3-dimensional spatial grid C, and $f(c) = 1$ within the ROI and zero in the rest of the scene. $\mathcal{X}_i$ can be identified/annotated by experts or using computational strategies, and is depicted via the color green in Fig. 1.We denote the final healthy atlas resulting from this process as $\mathcal{A} = (C, g)$. We define $g(c) = \frac{1}{N} \sum_N f(c) \in [0, 1]$, as the frequency of a particular location $c \in C$ with $f(c) = 1$ (i.e. corresponding to the specific ROI such as the rectal wall); across N different input subject scenes via the following 3 transformations[1]:

1. A simple transformation, τ_ρ, is used to map N different subject scenes $\mathcal{X}_i$ such that they are all centered and isotropically scaled in X, Y, and Z axes. The resulting initial atlas, $\mathcal{A}_\rho$, is therefore not dependent on selecting a specific subject as the template and is constructed by computing $\mathcal{A}_\rho = (C, g)$ for every location $c \in C$, across all N studies after τ_ρ has been applied, and $g(c) \in [0, 1]$ is the frequency of a location corresponding to the ROI.

2. Affine registration is used to compute τ_α for projecting all $\mathcal{X}_{\rho,i}$ onto $\mathcal{A}_\rho$. The affinely transformed subject scenes are used to construct $\mathcal{A}_\alpha = (C, g)$, based on recomputing $g(c)$ $\forall c \in C$, across all N studies.

3. Deformable registration is used to align $\mathcal{X}_{\alpha,i}$ to $\mathcal{A}_\alpha$. The final structural atlas $\mathcal{A} = (C, g)$, is constructed based on recomputing $g(c)$, $\forall c \in C$, across N deformed subject scenes.

Registration steps were implemented using the *elastix* registration tool[2]. Evaluation was done in terms of Dice overlap in rectal wall regions across all patients in S_1, after each atlas construction step (rigid, affine, deformable) where a higher Dice value was taken to imply a more accurate structural atlas representation. Additionally, the impact of the grid spacing parameter within the deformable registration step was varied between 7×7, 9×9 and 11×11 voxels to determine its impact on the final atlas representation.

Supplementary Figure S1 depicts a line plot of average Dice overlap in rectal wall regions between each patient in S_1 and the rectal atlas $\mathcal{A}$, after each construction step (coarse adjustment, rigid, affine and deformable registration). Atlas accuracy demonstrates a significant improvement at every stage of construction, from an initial Dice of 0.435 ± 0.127 (after coarse alignment) to a final Dice value of 0.881 ± 0.091. Qualitatively, the improvement in alignment of rectal wall regions and sequential refinement of the atlas can be observed in Supplementary Figure S2, based on a 3D rendering of the structural rectal atlas $\mathcal{A}$ generated after each stage of construction. While no significant differences are observed in atlas accuracy between different grid spacings, 7×7 can be seen to yield the highest average overlap (though only marginally so).

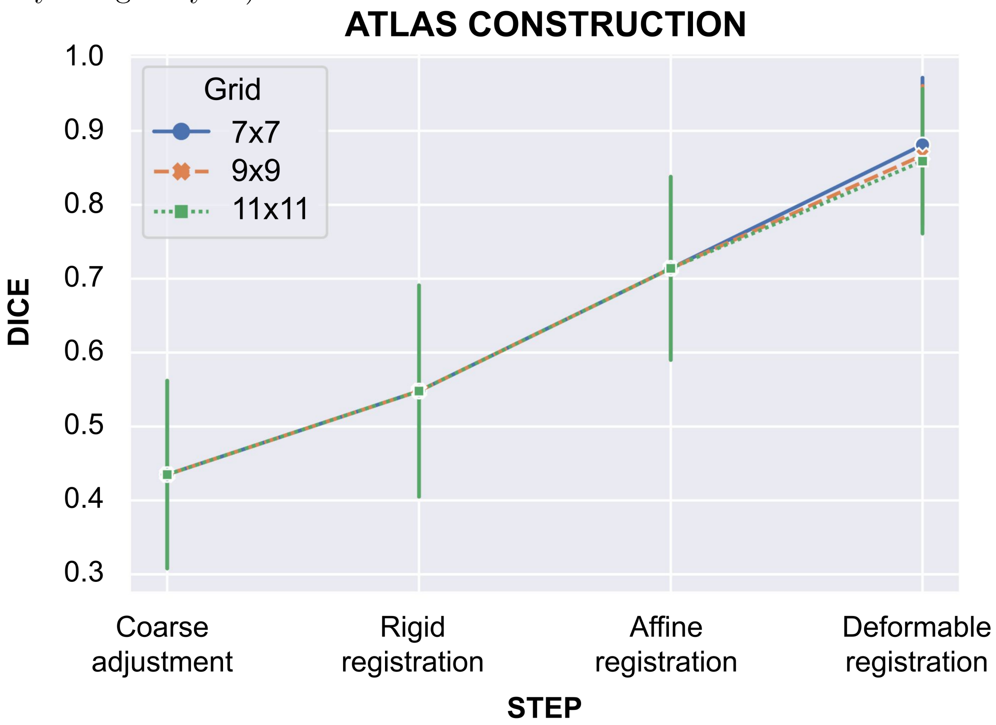

Figure S1: Line plot of atlas accuracy in terms of overall Dice similarity coefficient at each atlas construction step, where different colors correspond to different grid spacings.

Figure S3a further visualizes differences between the structural rectal atlas (green) and the rectal wall volume (yellow) for a representative ypT0-2 and a representative ypT3-4 rectal cancer patient. Note that in the patient with pathological stage regression (ypT0-2, top row), the overall rectal structure is largely aligned and similar in appearance to the atlas. By comparison, the ypT3-4 patient (bottom row) depicts markedly less alignment between the patient rectum and the atlas. Deformation fields obtained by registering these patient

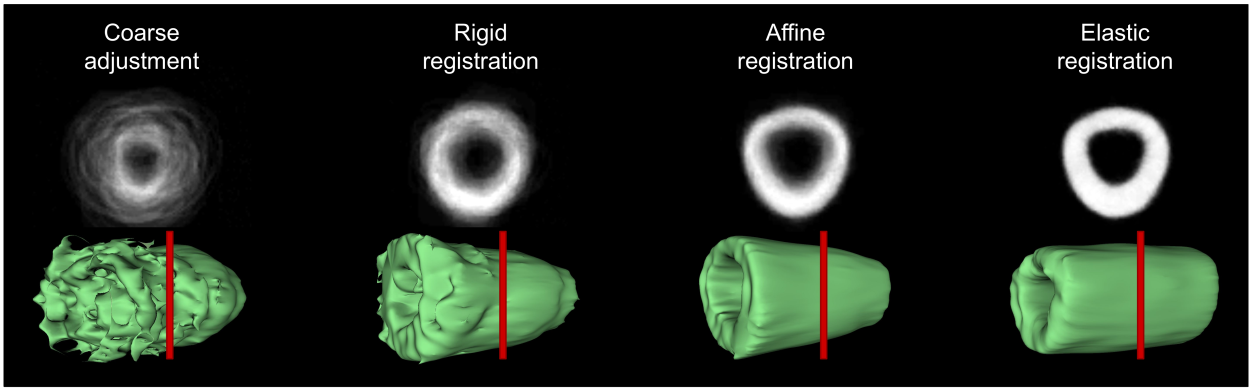

Figure S2: 3D rendering of structural rectal atlas after each stage of atlas construction. Note more consistent and refined visualization of the rectal wall at the final stage (deformable registration) compared to the noisy initial estimates (coarse alignment).

volumes to the atlas are visualized in Figure S3b , indicating both the magnitude (depicted as a heatmap) as well as orientation of the deformation field (as arrow vectors). Intuitively, more pronounced and chaotic deformations can be observed for the ypT3-4 patient compared to the ypT0-2 patient.

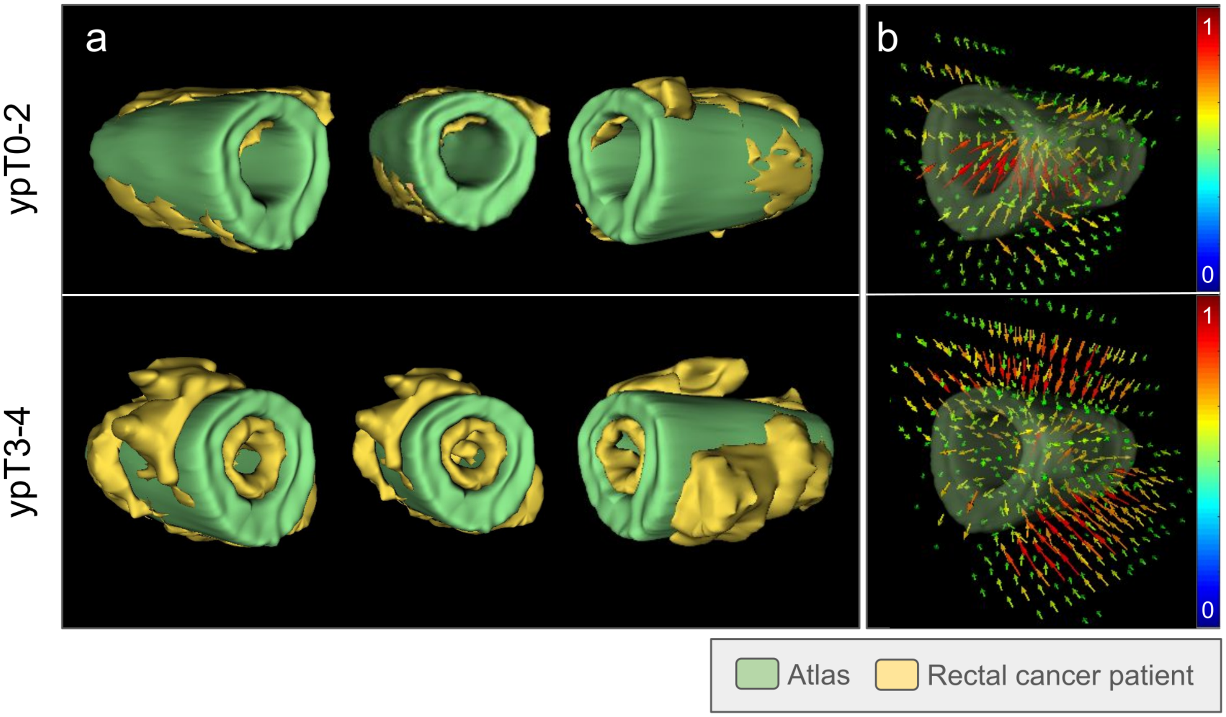

Figure S3: (a) 3D rendering of structural rectal atlas (in green) in conjunction with rectal wall volumes of rectal cancer patients (in yellow), aligned in the same anatomical space. (b) Visualization of deformation field vectors overlaid on the atlas for each case. Note that the top row represents a ypT0-2 rectal cancer patient, while the bottom row corresponds to ypT3–4 rectal tumor.

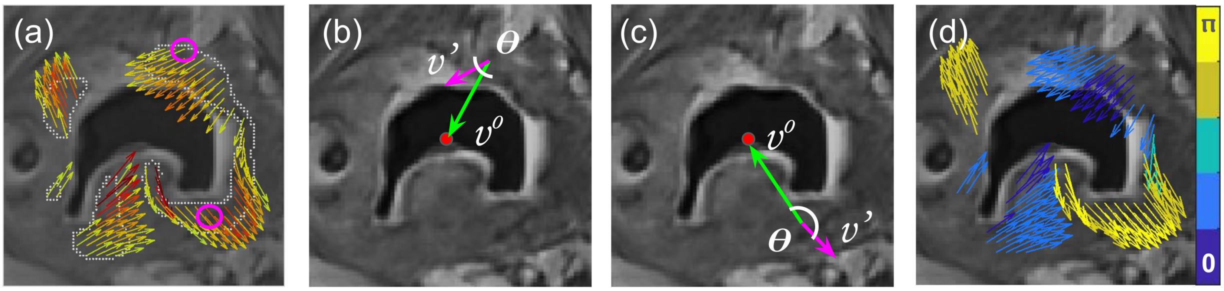

Figure S4: Computing deformation orientations, illustrated in the context of our rectal cancer use-case. (a) shows the regions with the largest deformations, identified via Markov random field (MRF)-based clustering. (b)-(c) depict two samples of angle calculation from (a). Magenta arrows in (b)-(c) illustrate inward and outward deformation vectors, respectively. Angle calculation utilizes a geometric arccos transform between the deformation vector v' (magenta arrows) and a vector v^o (green arrows) defined with respect to the lumen centroid (red point). (d) resultant angles are quantized into 5 bins (0-20°, 20-80°, 80-100°, 100-160°, and 160-180°) to yield the orientation descriptor $\mathbb{F}_o$.

Table S1: Summary of imaging parameters of multi-institution data cohort used in this study

	S_1	S_2		S_3
	**Inst. 1 UHCMC (n=75)**	**Inst. 1 UHCMC (n=61)**	**Inst. 2 CCF (n=27)**	**Inst. 3 PCC (n=19)**
**Imaging Parameters**				
In-plane Resolution (mm)	0.625 - 0.703	0.256 - 0.938	0.313 - 0.898	0.508 - 0.938
Slice Thickness (mm)	3.0 - 4.0	3.0 - 4.0	3.0 - 6.0	3.0 - 5.0
Field of view (px)	240 - 704 x 240 - 704 x 20 - 58	240 - 704 x 240 - 704 x 20 - 58	256 - 640 x 252 - 640 x 13 - 79	256 - 512 x 256 - 512 x 22 - 61
Repetition Time (ms)	5000 - 7200	3253 - 12880	3400 - 13333	3000 - 7430
Echo Time (ms)	96 - 100	67 - 110	84 - 166	81 - 88
Sequence	TSE	TSE	TSE	SE
Magnet Strength				
3 T	75	51	3	
1.5 T		10	24	19
Scanner				
Siemens Symphony		1	4	
Siemens Avanto		4	12	
Siemens Espree			3	
Siemens Aera		4	4	
Siemens Skyra	75		3	
Siemens Verio		40		
Philips Achieva			1	
Philips Med Ingenuity		5		
Philips Health Ingenia		7		
GE MS Signa HDxt				5
GE MS Signa Artist				14
Plane Axial Through Tumor				
Transverse	75	61	27	19
Gel use	No	Yes	Yes	No

Table S2: Symbols and notations employed in this paper

Symbol	Description
$\mathcal{X}_i = (C, f)$, $\forall i \in \{1, ..., N\}$	Subject scene i with $\forall c \in C,\ f(c) = 1$ represents rectal wall and $f(c) = 0$ the rest of the scene
$A_\rho = (C, g)$	Initial version of healthy rectal atlas after each $\mathcal{X}_i$ is centered and scaled, and $g(c) = \frac{1}{N}\sum_N f(c) \in [0, 1]$
$A_\alpha = (C, g)$	Second version of healthy rectal atlas after affine projection of all $\mathcal{X}_i$ onto A_ρ, and $g(c) = \frac{1}{N}\sum_N f(c) \in [0, 1]$ is recomputed
$\mathcal{A} = (C, g)$	Final version of healthy rectal atlas after deformable projection of all $\mathcal{X}_i$ onto A_α, and $g(c) = \frac{1}{N}\sum_N f(c) \in [0, 1]$
$\mathcal{I}$	Rectal wall annotated on rectal cancer patient MRI
$\bar{\mathcal{A}} = T(\mathcal{A}, \mathcal{I})$	$T(.)$ is the forward transformation that maps $\mathcal{A}$ to the rectal wall $\mathcal{I}$
$\mathcal{I}' = T^{-1}(\mathcal{I}, \mathcal{A})$	$T^{-1}(.)$ is the inverse transformation that maps $\mathcal{I}$ to the $\mathcal{A}$ space

Table S3: Classifier performance comparing $\mathbb{F}_{mo}$, $\mathbb{F}_t$, and $\mathbb{F}_{mto}$ in both 2D and 3D; via a *QDA* model. Mean (standard deviation) of each performance measure are reported across 50 runs of 3-fold cross validation in the discovery cohort and for hold-out evaluation in the validation cohort. Abbreviations: Feat set = feature set (number of features), AUC = area under the receiver ROC curve, Acc = accuracy, Sens = sensitivity, Spec = specificity, Prec = precision. * Indicates statistical significance based on p-value ≤ 0.02 w.r.t. $\mathbb{F}_{mto}$.

	**Feat set**	**$\mathbb{F}_{mo}$ (n=9)**		**$\mathbb{F}_t$ (n=4)**		**$\mathbb{F}_{mto}$ (n=11)**	
	**Cohort**	**Discov.**	**Valid.**	**Discov.**	**Valid.**	**Discov.**	**Valid.**
**2D**	**AUC**	0.73 (0.06)*	0.68	0.62 (0.09)*	0.57	0.79 (0.07)	0.67
	**Acc**	0.73 (0.04)*	0.63	0.67 (0.06)*	0.65	0.77 (0.04)	0.67
	**Sens**	0.57 (0.15)*	0.53	0.44 (0.23)*	0.47	0.69 (0.15)	0.53
	**Spec**	0.85 (0.08)	0.67	0.85 (0.12)	0.73	0.84 (0.08)	0.73
	**Fscore**	0.63 (0.12)*	0.47	0.5 (0.19)*	0.45	0.71 (0.08)	0.50
	**Prec**	0.77 (0.09)	0.42	0.7 (0.2)*	0.44	0.77 (0.07)	0.47
**3D**	**AUC**	0.71 (0.05)*	0.64	0.65 (0.08)*	0.56	0.84 (0.06)	0.65
	**Acc**	0.74 (0.04)*	0.60	0.69 (0.05)*	0.50	0.82 (0.04)	0.67
	**Sens**	0.71 (0.11)*	0.53	0.51 (0.21)*	0.40	0.76 (0.13)	0.47
	**Spec**	0.76 (0.08)*	0.64	0.83 (0.12)*	0.55	0.87 (0.07)	0.76
	**Fscore**	0.69 (0.06)*	0.46	0.56 (0.14)*	0.33	0.78 (0.07)	0.47
	**Prec**	0.69 (0.06)*	0.40	0.73 (0.14)*	0.29	0.82 (0.07)	0.47

Table S4: Classifier performance comparing $\mathbb{F}_{mo}$, $\mathbb{F}_t$, and $\mathbb{F}_{mto}$ in both 2D and 3D; via a *Random Forest* model. Mean (standard deviation) of each performance measure are reported across 50 runs of 3-fold cross validation in the discovery cohort and for hold-out evaluation in the validation cohort. Abbreviations: Feat set = feature set (number of features), AUC = area under the receiver ROC curve, Acc = accuracy, Sens = sensitivity, Spec = specificity, Prec = precision. * Indicates statistical significance based on p-value $\leq$ 0.02 w.r.t. $\mathbb{F}_{mto}$.

	**Feat set**	**$\mathbb{F}_{mo}$ (n=9)**		**$\mathbb{F}_t$ (n=4)**		**$\mathbb{F}_{mto}$ (n=11)**	
	**Cohort**	**Discov.**	**Valid.**	**Discov.**	**Valid.**	**Discov.**	**Valid.**
**2D**	**AUC**	0.80 (0.05)*	0.73	0.67 (0.07)*	0.73	0.86 (0.04)	0.73
	**Acc**	0.78 (0.04)*	0.67	0.69 (0.05)*	0.69	0.82 (0.03)	0.71
	**Sens**	0.75 (0.1)*	0.53	0.56 (0.24)*	0.20	0.85 (0.08)	0.53
	**Spec**	0.80 (0.09)	0.73	0.79 (0.16)	0.91	0.80 (0.08)	0.79
	**Fscore**	0.74 (0.05)*	0.50	0.58 (0.16)*	0.29	0.80 (0.04)	0.53
	**Prec**	0.75 (0.08)	0.47	0.73 (0.15)*	0.50	0.77 (0.07)	0.53
**3D**	**AUC**	0.80 (0.05)*	0.68	0.67 (0.08)*	0.58	0.88 (0.03)	0.67
	**Acc**	0.78 (0.04)*	0.67	0.70 (0.06)*	0.58	0.84 (0.04)	0.65
	**Sens**	0.84 (0.14)	0.40	0.50 (0.21)*	0.33	0.85 (0.09)	0.40
	**Spec**	0.73 (0.08)*	0.79	0.85 (0.15)	0.70	0.83 (0.07)	0.76
	**Fscore**	0.76 (0.07)	0.43	0.56 (0.15)*	0.33	0.82 (0.04)	0.41
	**Prec**	0.70 (0.05)*	0.46	0.76 (0.19)	0.33	0.80 (0.06)	0.43

Table S5: Classifier performance comparing $\mathbb{F}_{mto}$ in 3D when considering a subvolume of 5 slices or all available slices in the rectal volume; via a LDA model. Mean (standard deviation) of each performance measure are reported across 50 runs of 3-fold cross validation in the discovery cohort and for hold-out evaluation in the validation cohort. Abbreviations: AUC = area under the receiver ROC curve, Acc = accuracy, Sens = sensitivity, Spec = specificity, Prec = precision. * Indicates statistical significance based on p-value $\leq$ 0.02 between 5-slice subvolume and entire volume.

	F_{mto} (5 slices)		F_{mto} (all slices)	
	**Discovery**	**Validation**	**Discovery**	**Validation**
**AUC**	0.84 (0.04)*	0.66*	0.80 (0.06)	0.59
**Acc**	0.83 (0.03)*	0.65*	0.78 (0.05)	0.50
**Sens**	0.80 (0.08)*	0.53	0.68 (0.15)	0.53
**Spec**	0.85 (0.05)	0.70*	0.85 (0.10)	0.48
**Fscore**	0.80 (0.04)*	0.48*	0.72 (0.08)	0.40
**Prec**	0.80 (0.05)	0.44*	0.08 (0.11)	0.32

Table S6: Classifier performance using clinical TNM (cTNM) and rectal wall volume via an LDA model. Mean (standard deviation) of each performance measure are reported across 50 runs of 3-fold cross validation in the discovery cohort and for hold-out evaluation in the validation cohort. Abbreviations: AUC = area under the receiver ROC curve, Acc = accuracy, Sens = sensitivity, Spec = specificity, Prec = Precision.

**Feature set**	**cTNM**		**Rectal wall volume**	
**Cohort**	**Discovery (n=33)**	**Validation (n=46)**	**Discovery (n=40)**	**Validation (n=48)**
**AUC**	0.62 (0.07)	0.38	0.73 (0.03)	0.61
**Acc**	0.65 (0.04)	0.54	0.73 (0.03)	0.6
**Sens**	0.57 (0.2)	0.2	0.71 (0.12)	0.27
**Spec**	0.71 (0.14)	0.71	0.74 (0.12)	0.76
**Fscore**	0.57 (0.16)	0.22	0.68 (0.04)	0.3
**Prec**	0.67 (0.13)	0.25	0.68 (0.06)	0.33

Table S7: Evaluating StODeO descriptor robustness and reproducibility between groups defined based on magnetic field strength as well as scanners used for image acquisition. Median and inter-quartile range (IQR) of feature values are provided together with the p-value from Wilcoxon ranksum testing and instability scores (IS).

Feature Name	Magnetic Field Strength 1.5T (34)	Magnetic Field Strength 3T (54)	Scanner manufacturer Siemens (75)	Scanner manufacturer Philips (13)
Skewness Gabor XY	-0.87 (-1.82 - 1.2)	-0.41 (-1.13 - 0.95)	-0.26 (-1.32 - 1.18)	-0.81 (-1.79 - -0.03)
	p-value = 0.339, IS = 0.1		p-value = 0.151, IS = 0.04	
Median Magnitude Deformation	-0.42 (-1.52 - 0.82)	-0.54 (-1.4 - 0.63)	-0.52 (-1.41 - 0.89)	-0.54 (-1.56 - 0.16)
	p-value = 0.99, IS = 0.06		p-value = 0.452, IS = 0.08	
Skewness Gradient Sobel YX	0.12 (-0.86 - 0.66)	-0.09 (-0.95 - 1.3)	0.17 (-0.85 - 1.01)	-0.85 (-1.48 - 0.54)
	p-value = 0.54, IS = 0.12		p-value = 0.18, IS = 0.04	
Variance CoLlAGe idm ws=5	-0.25 (-1.05 - 1.08)	-0.29 (-1.09 - 0.99)	-0.23 (-0.98 - 1.08)	-0.97 (-1.39 - 0.55)
	p-value = 0.915, IS = 0.04		p-value = 0.173, IS = 0.08	
Std Dev Magnitude Deformation	-0.62 (-1.4 - 1.09)	-0.21 (-1.3 - 0.73)	-0.26 (-1.33 - 1.07)	-0.27 (-1.43 - 0.23)
	p-value = 0.8, IS = 0.04		p-value = 0.41, IS = 0.08	
Skewness CoLlAGe info1 ws=5	-0.17 (-0.84 - 0.94)	-0.2 (-1.08 - 1.17)	-0.2 (-0.99 - 0.93)	0.4 (-0.79 - 1.32)
	p-value = 0.867, IS = 0.08		p-value = 0.417, IS = 0.04	

References

[1] Rusu M, Bloch BN, Jaffe CC, Genega EM, Lenkinski RE, Rofsky NM, et al. Prostatome: A combined anatomical and disease based MRI atlas of the prostate. Medical Physics. 2014 jun;41(7):072301.

[2] Klein S, et al. elastix: a toolbox for intensity based medical image registration. IEEE Trans Med Img. 2010;29(1):196 205.
